# Management of the Lateral Walls in Crooked Nose

**Published:** 2012-07

**Authors:** Ahmet Seyhan

**Affiliations:** Department of Plastic Surgery, Celal Bayer University, Izmir, Turkey

**Keywords:** Rhinoplasty, Nasal cartilages, Nasal septum

## Abstract

In crooked noses, the lateral walls are different in shape and symmetry. Although the septum is very important to obtain a straight nose, identical and symmetrical lateral walls are needed for a straight looking nasal dorsum. As well as the septum, lateral walls also contribute in nasal skeletal support and stability. Thus, obtaining identical and symmetrical lateral walls is important. In order to obtain symmetrical and stable lateral walls, the requirements are to equalize the height, to set in a symmetric location and finally to stabilize the symmetry. These requirements must be taken into consideration while performing the steps of rhinoplasty, namely, hump resection, osteotomies and stabilization by spreader grafts or flaps. Here, we describe the management of the lateral walls in crooked nose in 7 cases.

## INTRODUCTION

In the crooked nose, the septal straightening is the first requirement of the surgery. The straightened septum may has have been weakened after these corrective interventions, and deviation or distortion can be expected in the late postoperative period.^1^,^2^ Thus the straightened septum must be supported by lateral walls on each side, to obtain long term stability. In the crooked nose, the lateral walls are different in shape and symmetry. Creating identical and symmetrical lateral walls are needed for a straight looking nasal dorsum, good nasal skeletal support and long term stability. Thus, obtaining identical and symmetrical lateral walls is important.

In order to obtain symmetrical and stable lateral walls, the requirements are to equalize the height, to set in inset a symmetric location and finally to stabilize the symmetry. These requirements must be taken into consideration while performing the steps of rhinoplasty, namely, hump resection, osteotomies and stabilization by spreader grafts or flaps. A combination of these mentioned procedures can be selected for each patient. The main principle is creating straight looking nasal dorsum and stable nasal skeleton, without unnecessarily weakening the skeletal components, the septum and lateral walls. Here we describe the management of the lateral walls in crooked nose in 7 cases.

## CASE REPORTS

**Fig. 1:**
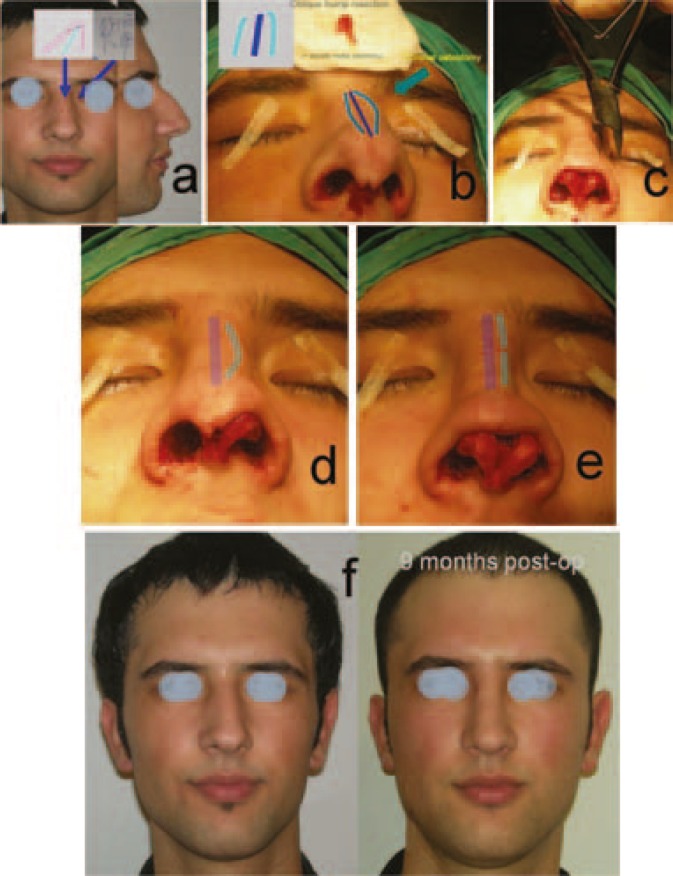
a) Preoperative views. b) After the oblique hump resection. c) A bone cutting forceps will be used for the osteotomy. d,e) Before and after the osteotomy. f) Preoperative and postoperative front views (Case 1).

**Fig. 2:**
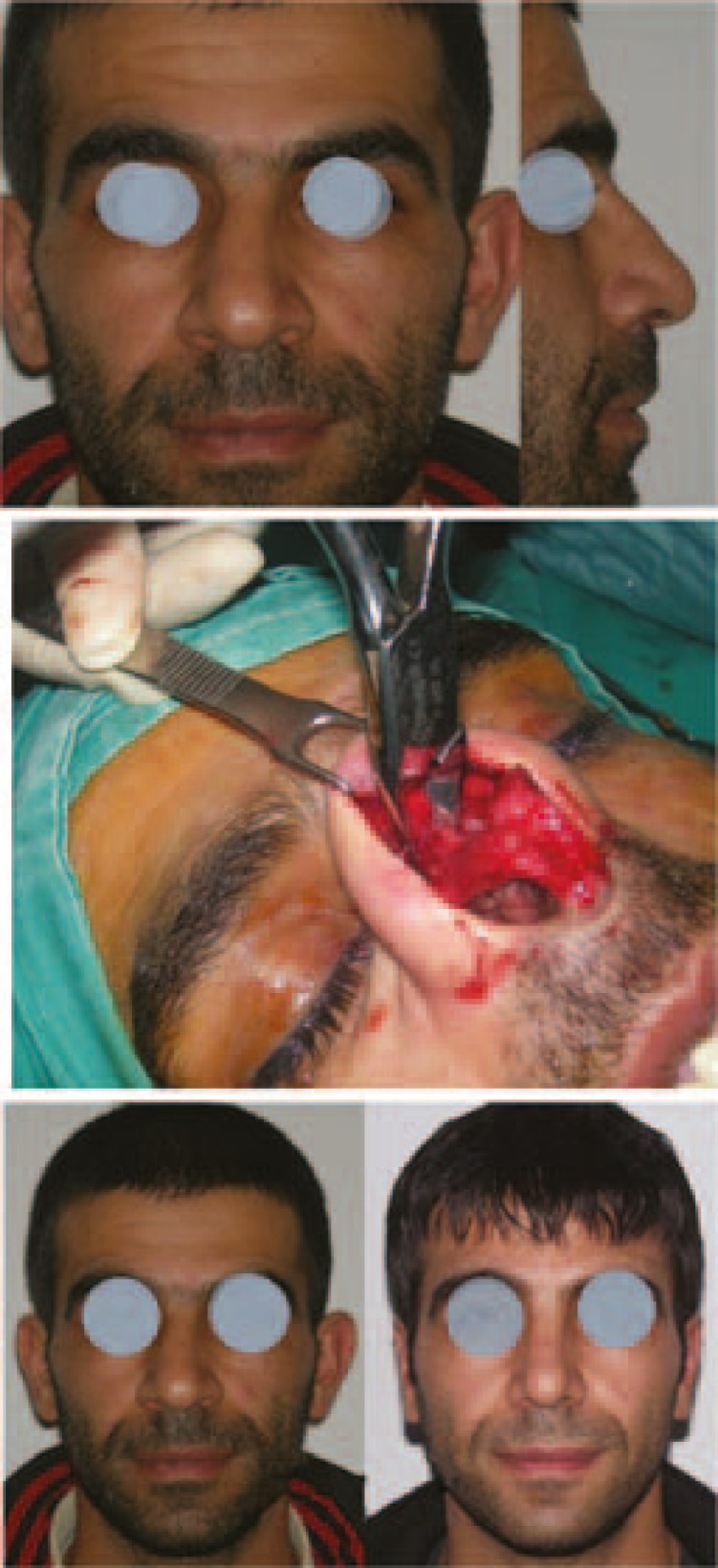
a) Preoperative views. b) During the coronal osteotomy. c) Preoperative and postoperative front views (Case 2).

**Fig. 3:**
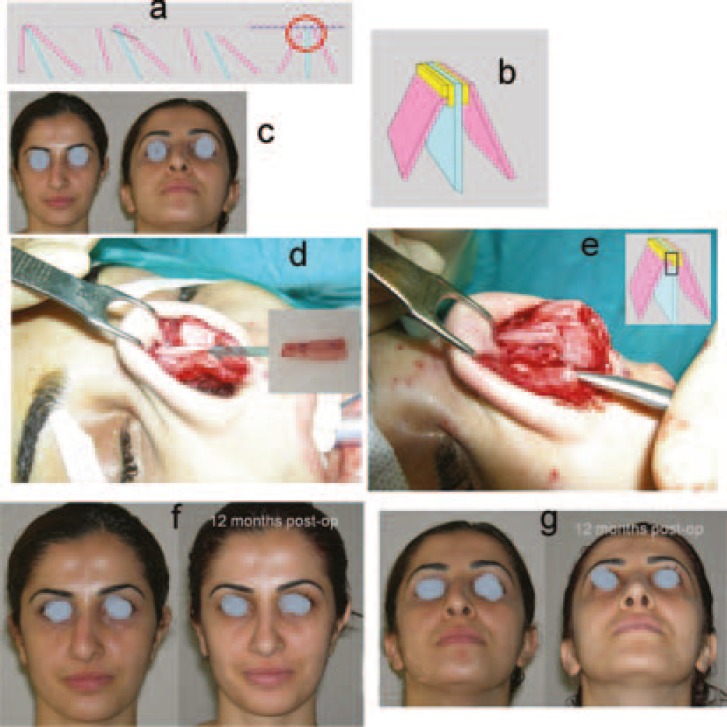
a) Complete saving the shorter wall with oblique hump resection, medial and lateral osteotomy on the shorter wall, out-fracture the shorter wall, removal the remainder hump of the septum and longer wall components, and centralizing the structures after the osteotomies for the longer wall. b) Drawing of the “Stepped spreader graft technique”. c) Preoperative views. d) The graft was prepared and e) put into its place. The edge of the lateral wall needs to fit properly into the stepped area of the graft. f) Before and after the operation. g) From below. *From* *“Seyhan* *A, Ozden* *S,* *Gungor* *M,* *at* *al.* *A double* *layered,* *stepped spreader graft for deviated nose. Ann Plast Surg. 62:604,* *2009* (Case 3)*.”*

**Fig. 4:**
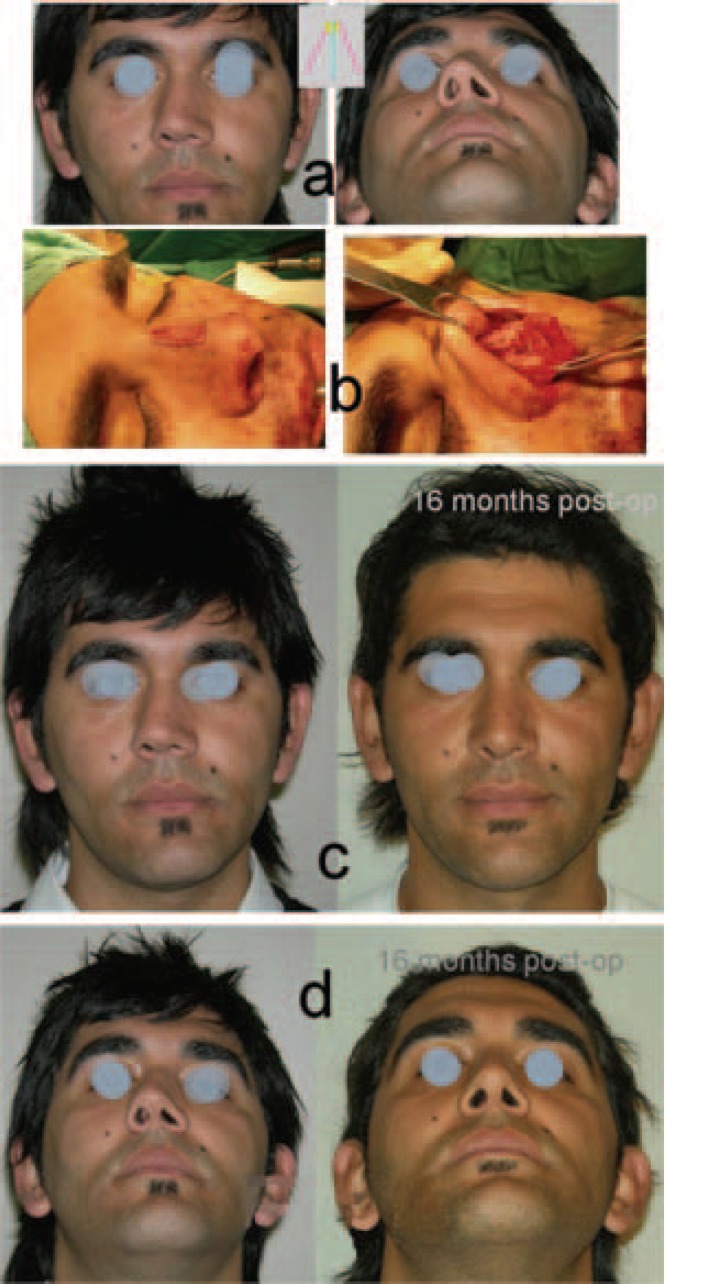
a) Preoperative views. b) The “stepped spreader graft”graft was prepared and put into its place. c) Before and after the operation. d) From below. *From* *“Seyhan* *A,* *Ozden* *S,* *Gungor* *M,* *at al.* *A* *double* *layered,* *stepped* *spreader* *graft for deviated nose. Ann Plast Surg. 62:604, 2009 *(Case 4)*.”*

**Fig. 5:**
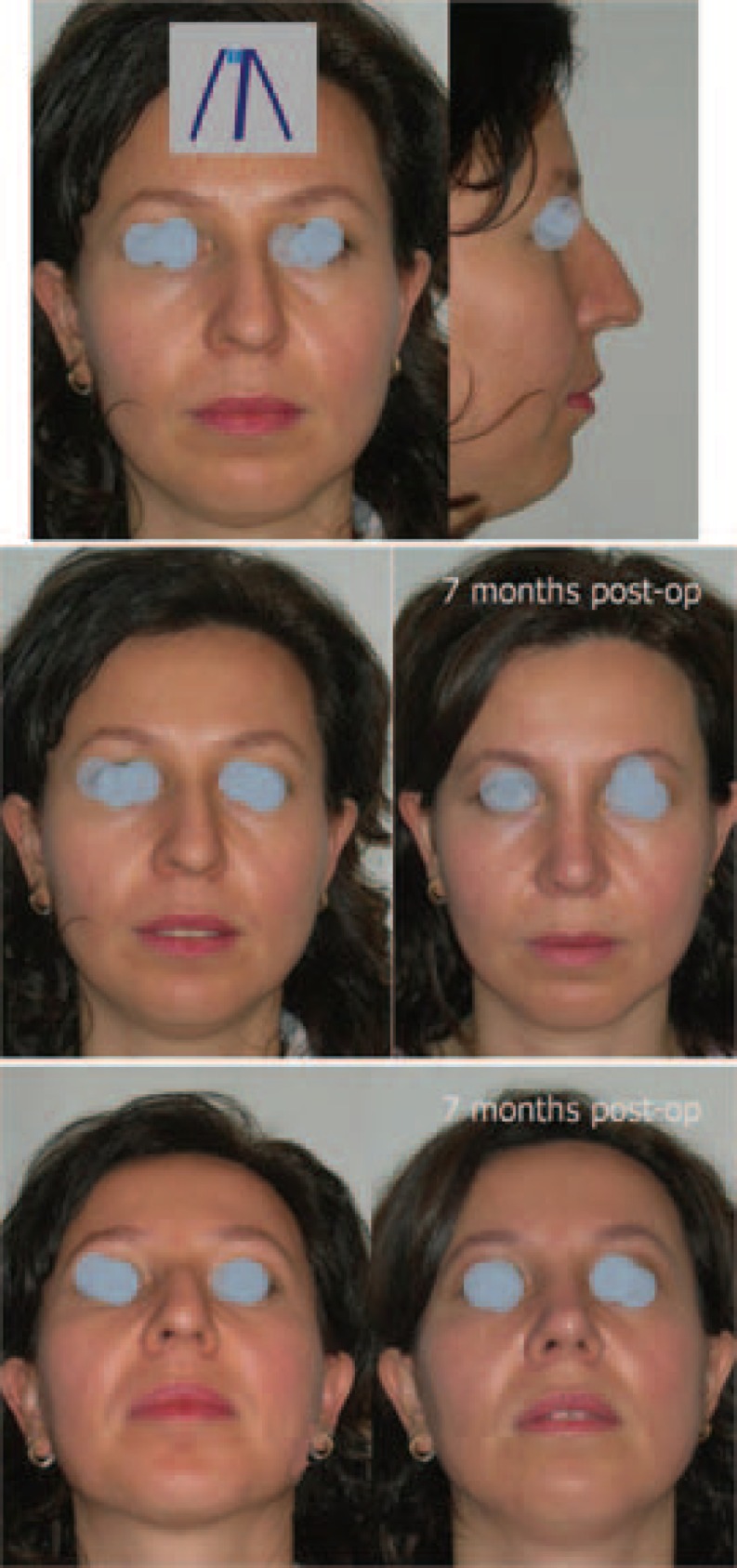
a) Preoperative views. b) Before and after the operation. c) From below (Case 5).

**Fig. 6:**
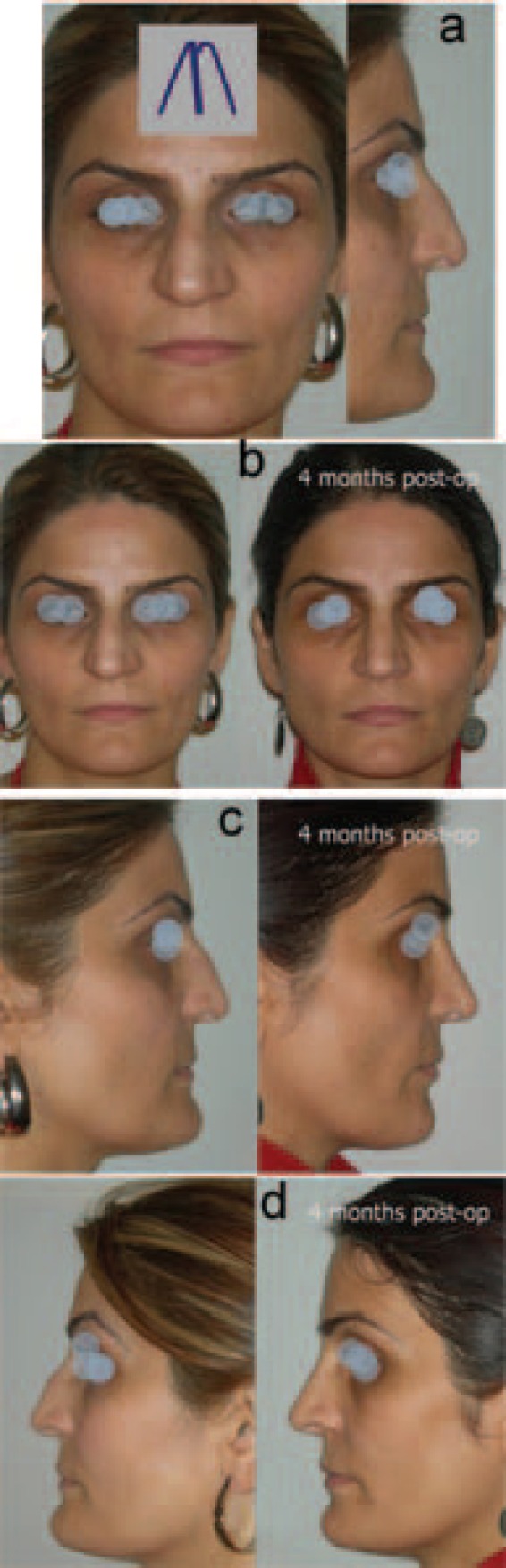
a) Preoperative views. b) Before and after the operation. c) From below (Case 6).

**Fig. 7:**
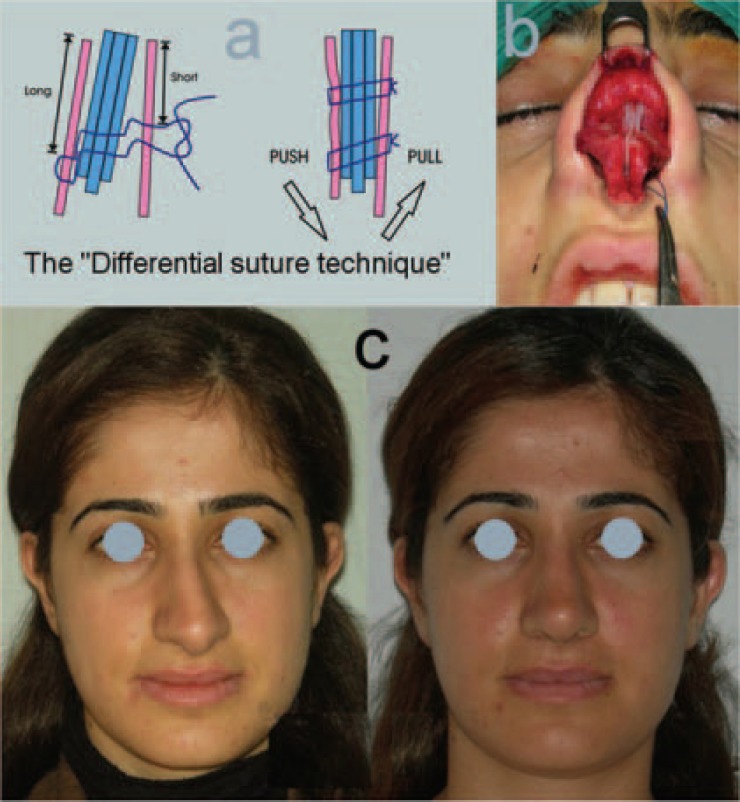
a) Drawing of the “Differential septal suture technique” *From* *“Seyhan* *A,* *Ozden* *S,* *Gungor* *M, at* *al.* *A double layered,* *stepped* *spreader graft for deviated* *nose.* *Ann* *Plast Surg.* *62:604,* *2009.”* b) Preoperative view. c) Before and after the operation (Case 7).

In case 1, oblique hump resection and coronal osteotomy were performed. After the oblique hump resection (osteotomies were performed), the left wall could not be pushed to the midline, because of its roundness. Therefore, a coronal (mid-transverse) osteotomy was performed. A coronal osteotomy was performed with a bone cutting forceps (Figure 1). In case 2, with the same procedure, an oblique hump resection and coronal osteotomy were performed (Figure 2). Case 3 is an example for the «Stepped spreader graft» procedure. The prepared SSG was placed on the right side of the septum. The edge of the lateral wall was needed to fit properly into the stepped area of the graft (Figure 3). Case 4 is another example for the «Stepped spreader graft» procedure. The stepped spreader graft was prepared and attached to the septum (Figure 4). In case 5, two spreader grafts were placed on the right side (Figure 5). In case 6, only a left sided spreader flap was performed (Figure 6) and in case 7, the “differential suture technique” was used (Figure 7).

## DISCUSSION

Septal straightening procedures are out of the scope of this paper. Instead, (not all but) commonly required and useful procedures regarding the lateral walls are discussed in this paper. The main requirements of the correction of the crooked nose:

*(i) Equalizing the heights of the lateral walls*

*Shortening the longer wall: *To equalize the height, shortening the longer wall or lengthening the shorter wall is needed. If a considerable amount of dorsal height exists, shortening the longer wall is enough. This can be simply provided by an “*oblique hump resection”*. This is a well-known technique among plastic surgeons, and a beveled osteotomy during hump resection is performed (Figure 1). A more complete saving the shorter wall height is possible making firstly medial and lateral osteotomy on the shorter wall, and then out-fracture the shorter wall, then removal the remainder hump of the septum and longer wall components (Figure 2). Osteotomies for the longer wall can be performed, if needed.

*Lengthening the shorter wall: *Only an oblique hump resection may not be enough, when the shorter wall does not reach the planned dorsal level after the osteotomies. In order to lengthen the shorter wall, the “*Stepped spreader graft” *technique can be used (Figure 3).^3^

*(ii) Setting in a symmetric location*

After an oblique hump resection, in some cases, the remaining part of the longer wall can be kept in place. In this case, pushing the dorsal border of the shorter wall towards the septum is sufficient to obtain symmetry, keeping the base as stable as possible. Osteotomies in which a medial oblique dominant and an adjunctive lateral osteotomy can provide such an lateral wall movemet.^4^,^5^ Another problem may be an excess lateral roundness on one of lateral wall. As well as the asymmetrical apprearence, this roundness may also prevent the medialization of this lateral walls towards septum. An “*additional coronal or diagonal osteotomy*” can solve this problem.^6^

*(iii) Stabilizing the symmetry*

Created identical lateral walls must be stabilized in accordance to the midline. The septum slightly out of midline, one sided spreader graft or spreader flap, or bilateral spreader grafts with different thickness can be used. This stabilizes the lateral walls symmetrically according to the midline, without disturbing the septum. In that case, the undisturbed septum gives a strength and stability to the lateral walls and nasal skeleton.^7^ After the required osteotomies, if the septum, or even lateral walls tend to locate one side, a differential suture, between the septum and lateral walls can be used to bring the components to the midline and stabilize them.^8^ A combination of the required techniques is selected for each case.

In conclusion, Identical and symmetrical lateral walls can be created and stabilized with properly selected procedures mentioned in this article.

## CONFLICT OF INTEREST

The authors declare no conflict of interest.
